# Design and Characterization of a Screw Extrusion Hot-End for Fused Deposition Modeling

**DOI:** 10.3390/molecules26030590

**Published:** 2021-01-23

**Authors:** Tim Feuerbach, Markus Thommes

**Affiliations:** Laboratory of Solids Process Engineering, Department of Biochemical and Chemical Engineering, TU Dortmund University, Emil-Figge-Str. 68, 44227 Dortmund, Germany; tim.feuerbach@tu-dortmund.de

**Keywords:** 3D printing, fused deposition modeling, filament, extrusion, hot-end, screw

## Abstract

The filament is the most widespread feedstock material form used for fused deposition modeling printers. Filaments must be manufactured with tight dimensional tolerances, both to be processable in the hot-end and to obtain printed objects of high quality. The ability to successfully feed the filament into the printer is also related to the mechanical properties of the filament, which are often insufficient for pharmaceutically relevant excipients. In the scope of this work, an 8 mm single screw hot-end was designed and characterized, which allows direct printing of materials from their powder form and does not require an intermediate filament. The capability of the hot-end to increase the range of applicable excipients to fused deposition modeling was demonstrated by processing and printing several excipients that are not suitable for fused deposition modeling in their filament forms, such as ethylene vinyl acetate and poly(1-vinylpyrrolidone-co-vinyl acetate). The conveying characteristic of the screw was investigated experimentally with all materials and was in agreement with an established model from literature. The complete design information, such as the screw geometry and the hot-end dimensions, is provided in this work.

## 1. Introduction

Fused deposition modeling (FDM) is a widespread manufacturing technique in numerous pharmaceutical and medical applications, such as solid dosage forms [1,2], implants [3,4] and tissue engineering [5,6]. In FDM, thermoplastic materials are plasticized, extruded through a nozzle and deposited on a build platform to build three-dimensional objects layer by layer [7]. The feedstock material is provided in filament form and is usually produced by hot-melt extrusion in a preceding step [8]. In this way, filaments consisting of pharmaceutical excipients and active pharmaceutical ingredients can be manufactured in various combinations and compositions [9,10]. However, the range of filaments useable in the printing process is limited by the geometrical and mechanical properties of the individual filaments [11]. FDM hot-ends are typically designed for standard filament diameters of 1.75 mm and 3 mm [12], which must have tight tolerances to prevent clogging of the filament in the hot-end [13]. The nominal filament diameter is usually stored in the printer software to determine the filament feed velocity required to obtain a specific extrudate mass flow. Since there is no feedback control in most FDM printers, deviations from the nominal filament diameter lead directly to inconsistent flowrates, affecting the product quality [14]. In addition to the geometrical requirements of the filament, there are also mechanical requirements that must be met. In the process of pulling the filament from the storage spool, sufficient mechanical strength is necessary to avoid deformation or breaking of the filament [10,11]. Additionally, the subsequent pushing process into the hot-end frequently causes flexible filament materials to buckle and brittle filament materials to fracture [15].

The aims of this study were the development and characterization of an FDM hot-end based on single-screw extrusion to directly print pharmaceutical excipients from their powder or pellet forms without the need for an intermediate filament. The ability to directly print from powder eliminates the filament manufacturing step entirely, which reduces the amount of equipment required. Moreover, the range of materials applicable to FDM is extended, because the suitability of a material for the printing process is not limited by the mechanical properties in its filament form or the performance of the filament extrusion process and the tight filament diameter tolerance requirements.

In this study, the developed single-screw hot-end and the rationale of its design process are presented. The information provided is intended to provide a basis for the reader to be able to construct similar or modified versions of the presented hot-end. Results from characterization experiments of the equipment are shown, such as the determination of the residence time distribution of the material in the hot-end. Different materials that are either not suitable for FDM or are difficult to handle in their filament form were printed to demonstrate the capability of the hot-end to extend the range of pharmaceutical excipients applicable to FDM.

## 2. Materials and Methods

### 2.1. Materials

Poly(lactic-co-glycolic acid) (PLGA) with a monomer ratio of 85 mole % D,L-lactide and 15 mole% glycolide and dimethylaminoethyl methacrylate-copolymer (EPO) were provided by Evonik (Resomer RG 858 S and Eudragit E PO, Evonik Nutrition and Care GmbH, Darmstadt, Germany). Ethylene vinyl acetate (EVA) with a vinyl acetate content of 28 mole% was provided by Celanese (VitalDose, Celanese Production Germany GmbH and Co. KG, Frankfurt am Main, Germany). Poly(1-vinylpyrrolidone-co-vinyl acetate) (PVPVA) was provided by Ashland (Plasdone S-630, Ashland Industries Europe GmbH, Schaffhausen, Switzerland). Polyethylene glycol (PEG) with a mean molecular weight of 3350 was provided by Clariant (Polyglykol 3350 S, Clariant Produkte GmbH, Frankfurt am Main, Germany) and theophylline was provided by BASF (theophylline anhydrous powder, BASF, Ludwigshafen, Germany). All materials were used as received except for Resomer^®^ RG 858 S, which was dry granulated with a rotary tablet press (102i, Fette Compacting GmbH, Schwarzenbek, Germany) and ground in a glass blender (Kenwood Major Titanium with AT358 attachment, Kenwood Ltd., Havant Hampshire, United Kingdom) to improve its flowability.

### 2.2. Methods

#### 2.2.1. Hot-End Design

**Figure 1:**
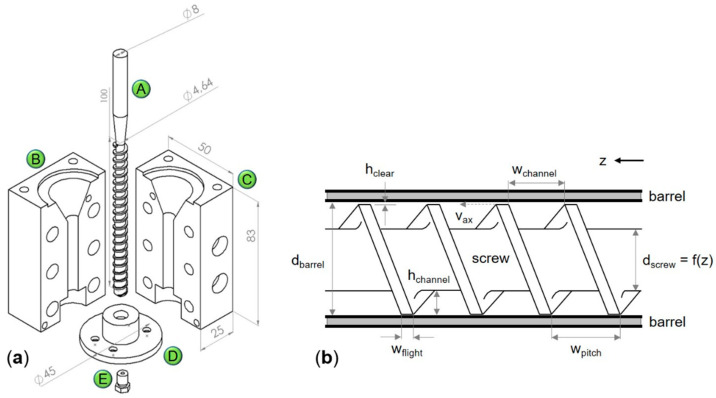
Drawing of the hot-end (**a**): (A) screw, (B) first barrel part, (C) second barrel part, (D) nozzle mounting and (E) printer nozzle. Sketch of the screw geometry (**b**).

Drawings of the individual hot-end components and the extruder screw geometry are shown in Figure 1. The hot-end consists of a custom two-part barrel, a nozzle mounting, a printer nozzle and a tapered extruder screw. The barrel, manufactured from 1.2344 steel, was heated by two heating cartridges (HZP-320W-8-100-24V, OTOM Group GmbH, Braeunlingen, Germany) and the extrusion temperature was measured with a PT 100 sensor (EF8G-PT100A-1.0-2L, OTOM Group GmbH, Braeunlingen, Germany). A commercial 400 µm nozzle (E3D v6, E3D Online, Chalgrove, United Kingdom) was used in this study and was connected to the barrel via the custom nozzle mounting.

The dimensions and geometrical design parameters of the extruder screw shown in Figure 1 are presented in Table 1. The extruder screw was manufactured from 1.7225 steel and was driven by a stepper motor (QSH6018-45-28-110, Trinamic Motion Control GmbH and Co. KG, Hamburg, Germany). In general, the geometrical design of an extruder offers several degrees of freedom. In this work in particular, the design of the extruder was focused on the feeding section. In order to ensure a successful material intake, it was necessary to provide sufficient free volume between the extruder screw and the extruder barrel. 

#### 2.2.2. Thermal Characterization

Differential scanning calorimetry (DSC Q2000, TA Instruments, New Castle, DE, USA) was used to determine the glass transition temperatures and the melting temperatures of the utilized materials to obtain a first estimate of the required processing temperature in the printer hot-end. For each measurement, samples of approximately 5 mg were weighed with an analytical scale (AC210S, Sartorius, Goettingen, Germany) and a heat-cool-heat cycle with a heating rate of 10 K min^−1^ was performed in a nitrogen atmosphere. The glass transition temperature was determined in the second heating ramp, using the inflection point method.

#### 2.2.3. Material Processability

For the amorphous materials, the minimum extrusion temperature was determined by incrementally increasing the extrusion temperature from 40 °C above the measured glass transition temperature [16] until extrusion of the respective material was successful. For the crystalline and semi-crystalline materials, the temperature was gradually increased from 10 °C below the melting temperature until extrusion was successful.

#### 2.2.4. Conveying Characteristic

The conveying characteristic of the utilized screw was determined gravimetrically by extruding material at different screw speeds, collecting the material for a defined amount of time and weighing the collected material with an analytical scale (AC210S, Sartorius, Goettingen, Germany). The obtained mass flow was converted into the volumetric flow rate with the material density provided by the manufacturers.

The experimentally determined conveying characteristics were compared to a model that predicts the conveying characteristic of a single screw based on its geometry and the screw speed (adapted from: [17]), given by

(1) $$\dot{m}=\frac{1}{2}\;N_{\mathrm{flight}}\;\rho_{\mathrm{melt}}\;v_{\mathrm{axial}}\;w_{\mathrm{channel}}\;h_{\mathrm{channel}}\;F$$

where $\dot{m}$ is the extrudate mass flow, N_flight_ the number of parallel flights, ρ_melt_ the melt density, v_axial_ the axial component of the screw flight velocity at the barrel wall, w_channel_ the channel width, h_channel_ the mean distance from the screw root to the barrel wall and F a shape factor (see also Figure 1b). The axial velocity component is given by

(2) $$v_{\mathrm{ax}}{\;=\;\pi \;n\;d}_{\mathrm{barrel}}\;\cos\;\theta$$

where n is the rotational speed of the screw and d_barrel_ is the inner diameter of the barrel. θ is the helix angle at the barrel given by

(3) $$\theta \;=\;\arctan\left(\frac{w_{\mathrm{pitch}}}{{\pi \;d}_{\mathrm{barrel}}}\right)$$

where w_pitch_ is the pitch of the extruder screw. The model takes into account the axially conveyed volume based on the screw geometry and the rotational speed of the screw, but neglects backflow of the material due to the pressure build up at the nozzle. Based on the rather low mass flows obtained, the pressure gradient at the nozzle and the consequent backflow were assumed to be negligible. For each material, the screw speed range was determined, for which the linear correlation between screw speed and volumetric flow rate was still sufficient at the previously determined minimum extrusion temperature.

#### 2.2.5. Residence Time Distribution

Residence time measurements were conducted during extrusion of PVPVA using theophylline as the tracer material [18]. The experiments were conducted at the minimum extrusion temperature for PVPVA of 165 °C and a mass flow of 15 mg min^−1^. 2 mg of theophylline powder were fed into the hot-end as the impulse signal. The extrudate was collected and discretized into compartments equal to one second of residence time. The compartments were subsequently dissolved in demineralized water and the amount of dissolved theophylline was determined by UV/VIS spectroscopy (Jenway 7305 Spectrophotometer, Bibby Scientific Ltd., Staffordshire, UK) at a wavelength of 272 nm to determine the cumulative residence time distribution. The obtained mean residence times were compared to the theoretically determined hydrodynamic residence time, given by

(4) $$\tau =\frac{V}{\dot{V}},$$

where is the free volume available to the material, V, and the volumetric flow rate, $\dot{V}$.

#### 2.2.6. Printing

The hot-end was used in combination with an in-house developed FDM printer [19]. Cuboids with an edge length of 10 mm and a height of 4.8 mm were printed as a reference geometry to demonstrate the capability of the hot-end to print objects from materials that cannot be manufactured as suitable filament materials for FDM. The printed materials and the respective processing conditions are shown in Table 2. A layer height of 400 µm, a nozzle with a diameter of 400 µm and a printing speed of 0.5 mm s^−1^ were used throughout the study.

## 3. Results and Discussion

### 3.1. Hot-End

The hot-end was designed as a single-screw extruder for a custom FDM printer [19]. In contrast to a twin-screw extruder, a single-screw extruder has a linear relationship between screw speed and extrudate mass flow, provided that the available motor torque is sufficient to process the material under the given conditions. Moreover, the extrudate mass flow is independent of the performance of the powder feeder, which can be challenging, especially at low scales. However, the mixing capability of single-screw extruders without specific mixing zones is limited, making it necessary to blend the powders to be printed prior to the printing process. The utilized extruder screw has a linearly increasing core diameter to compress the material over the length of the screw and to build up the pressure required to extrude the material through the printer nozzle. For the agitation of the screw, a stepper motor was selected to be able to rotate the screw accurately in small increments to achieve consistent mass flows for the printing process.

The barrel was designed in two parts to simplify the cleaning process and thereby facilitate the changing of printing materials. The barrel was manufactured from a harder steel than the screw to prevent any damage to the barrel in case of incorrect alignment of the screw and barrel. The PT100 temperature sensor was installed close to the printer nozzle, and the measured temperature was used by a custom PI temperature controller to maintain the set extrusion temperature by adjusting the effective voltage provided to the heating cartridges. The custom nozzle mounting has an M6 thread that allows utilization and modular exchange of common commercial printer nozzles and custom nozzles.

### 3.2. Material Selection and Processability

Several materials that are not useable in filament form were selected for this study with the goal of demonstrating the ability of the presented hot-end to extend the range of materials applicable to FDM. EVA is a material that can exhibit high elasticity depending on the monomer ratio and the molecular weight, and thereby potentially lacks the rigidity required for feeding the filament into the printer hot-end without bending or buckling. The EVA material used in this study was reported as unsuitable filament material in the literature [20]. PVPVA and EPO are brittle materials, which in filament form frequently fracture during processing. For these materials also, unsuccessful processing in their filament form was found in the literature [21,22]. With lower molecular weights, PEG exhibits wax-like behavior and has restrictions similar to the aforementioned brittle materials. In the literature, successfully processed filaments were only found for PEG materials with higher molecular weights [23]. The utilized PLGA grade was the exception and was successfully processed in filament form [4], so it was used as a reference. Thermal characterization of the different materials was conducted to obtain a first estimate of the required processing temperature in the printer hot-end.

**Figure 2:**
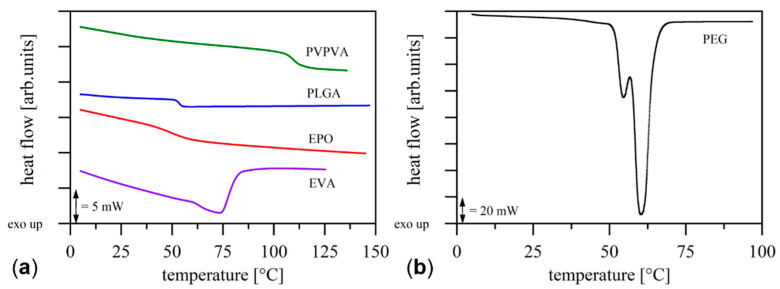
Representative second heating ramps of the differential scanning calorimetry measurements with the amorphous (**a**) and crystalline (**b**) materials in powder form.

The results of the differential scanning calorimetry measurements are shown in Figure 2. The representative second heating ramps indicate completely amorphous materials except for PEG and EVA, which were in crystalline and semi-crystalline form, respectively.

For the first extrusion experiments of the amorphous materials in the printer hot-end, the determined glass transition temperatures were used to estimate the required processing temperatures at 40 °C above the respective glass transition temperatures [16]. Starting from these estimated temperatures, the minimum extrusion temperatures were determined by incrementally increasing the extrusion temperature until processing of the respective material was successful. For EVA and PEG, the temperature was gradually increased from 10 °C below the melting temperature until material extrusion was successful. The experimentally determined minimum extrusion temperatures for the respective materials are shown in Table 3.

All utilized materials were processable in the hot-end. For the crystalline PEG and the semi-crystalline EVA, extrusion was not possible until close to the melting point, where a considerable decrease in viscosity occurred. The amorphous materials were processable in the range of 50 to 100 °C above their glass transition temperatures, depending on their individual rheological properties.

### 3.3. Conveying Characteristic and Printing

**Figure 3:**
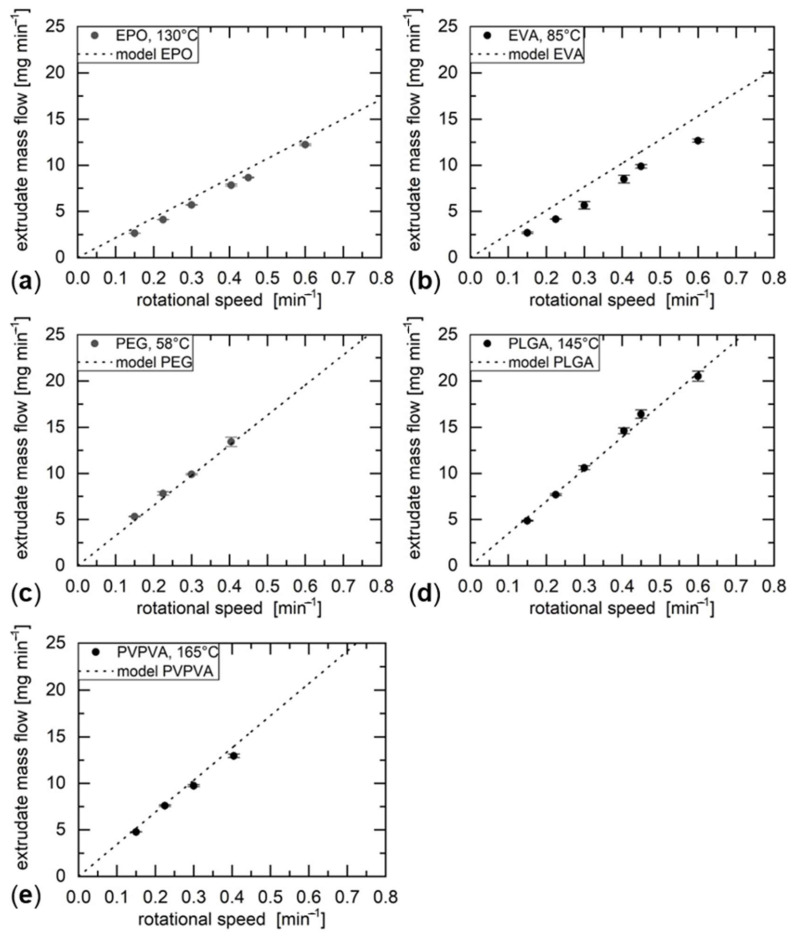
Measured conveying characteristics compared with the model for the investigated materials ((**a**) EPO, (**b**) EVA, (**c**) PEG, (**d**) PLGA, (**e**) PVPVA) at their respective minimum extrusion temperatures.

The linear range of the conveying characteristic was determined for each material at the respective minimum extrusion temperature and compared to a model (see Equation (1)) for the conveying characteristic of the screw utilized. The results are shown in Figure 3. In general, the experimental results were in agreement with the predicted conveying characteristics, supporting the suitability of the model utilized and the validity of the assumed negligible pressure-induced back flows of material. The achievable upper end of the linear conveying characteristic was different for the each of the investigated materials and dependent on the rheological properties of the materials at the respective temperatures. The upper end of the linear conveying characteristic is extendable by increasing the extrusion temperature, but was not considered in this work, since the achieved mass flows were already in the target range for the printer utilized.

A systematic deviation from the model was observed for EVA. Based on the elastic behavior of the material, it is likely that part of the energy transferred to the material was used for elastic recovery after the extrusion process at the expense of throughput. The observation of swelling of EVA extrudate strands during the experiments supported this assumption.

**Figure 4:**
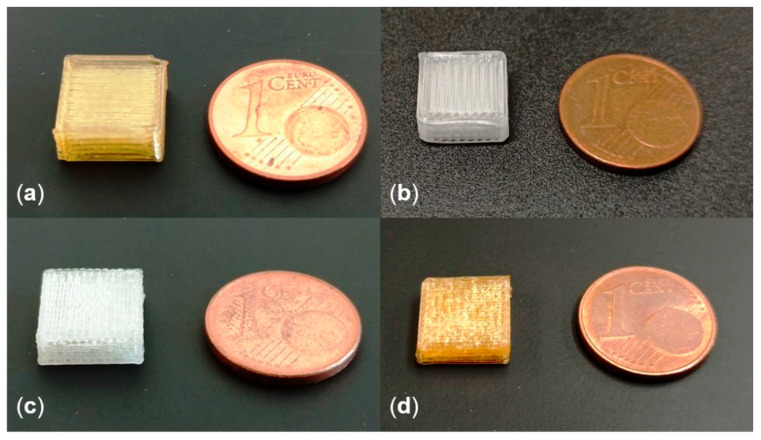
Successfully printed cuboids for EPO (**a**), EVA (**b**), PLGA (**c**) and PVPVA (**d**).

Despite the successfully determined conveying characteristic, the general processability of PEG was inconsistent due to the low molecular weight of the utilized grade and its crystallinity. The viscosity in the crystalline form was too high to be processable in the hot-end, whereas the viscosity in the molten state was too low to form a solid object after extrusion. The operating point close to the melting temperature of PEG resulted in temporarily successful extrusion that was nevertheless prone to frequent interruptions, due to local crystallization of PEG in the hot-end. For this reason, no proof-of-concept cuboid could be printed for PEG. The printed cuboids of the other materials are shown in Figure 4.

The utilized printing parameters for the different materials are presented in Table 2. The successful proof-of-concept prints with the materials utilized in this study show the capability of the developed hot-end to extend the range of applicable pharmaceutical excipients to FDM, which offers new opportunities for 3D printed pharmaceutical products.

### 3.4. Residence Time Distribution

The measurements of the residence time distribution were conducted with PVPVA and theophylline as a tracer material at the previously determined minimum extrusion temperature for PVPVA of 165 °C with a mass flow of 15 mg min^−1^. The obtained data are shown in Table 4.

The theoretical hydrodynamic residence time describes the residence time of the material in the absence of backmixing and is in agreement with the experimentally found values. However, based on the data obtained from the determined cumulative residence time distributions, it can be concluded that the material experiences backmixing inside of the hot-end. The backmixing was described by the dimensionless Bodenstein number, which is a measure for the backmixing characteristic of a system. The rather low value of 1.1 is representative of flow conditions similar to those in a continuously stirred tank, indicating high backmixing inside of the screw hot-end. Recently, it was shown that considerable backmixing is caused by the utilized commercial printer nozzle [19].

The absolute residence time values are considerably higher in comparison to typical extrusion processes and represent a potential risk for undesired thermal degradation of the material inside of the hot-end. There are several factors that contributed to the residence times obtained. The first factor is the design of the hot-end, which was designed for successful intake of feed material and thereby required sufficient free volume for the material. The second factor is the low required rotational speed of the extruder screw in this particular setup, caused by the limited displacement speed of the utilized build platform. The third factor is the additional free volume for the material that is provided by the nozzle mounting. The interaction of these factors leads to the considerably high residence time of the material in the hot-end. For future designs, the residence time can be substantially reduced by utilizing a different build platform that allows running the extrusion process at higher screw speeds, and by reducing the free volume inside of the hot-end.

## 4. Conclusions

A single-screw extrusion hot-end for fused deposition modeling was designed and characterized. The transparent illustration of the hot-end design is intended to provide a basis for similar or modified hot-ends tailored to specific materials or products. The presented single-screw extrusion hot-end processes material is used in powder form and does not require an intermediate filament. Several difficulties associated with filaments are thereby completely avoided, such as additional thermal exposure or mechanical and geometrical requirements. Several pharmaceutical excipients that are not suitable in filament form were successfully processed in the hot-end, and proof-of-concept prints were conducted. The conveying characteristic of the extruder screw was determined by gravimetrical measurements and was in agreement with an established model of axial conveying based on the screw geometry. The presented hot-end, which eliminates the need for prefabricated filaments, greatly extends the range of pharmaceutical-relevant excipients applicable to fused deposition modeling and offers new opportunities for 3D printed pharmaceutical products.

## Figures and Tables

**Table 1:** Dimensions of the extruder screw.

Symbol	Description	Value
d_barrel_	inner diameter of the barrel	8.02 mm
d_screw_	core diameter of the tapered extruder screw	4.64–6.80 mm
f(z)	slope of the tapered extruder screw	0.022 mm / mm
h_channel_	mean distance from the screw root to the barrel wall	1.15 mm
h_clear_	clearance between barrel and extruder screw	0.01 mm
N_flight_	number of parallel flights	1
w_channel_	channel width	4.00 mm
w_flight_	flight width	1.00 mm
w_pitch_	pitch of the extruder screw	5.00 mm

**Table 2:** Processing parameters used for the proof-of-concept prints of the different materials.

Material	Printing Temperature [°C]	Extrudate Mass Flow [mg min^−1^]	Rotational Speed [min^−1^]	Cuboid Printed?
EPO	130	4.0	0.225	yes
EVA	85	3.8	0.225	yes
PEG	58	4.9	0.150	no
PLGA	145	5.2	0.150	yes
PVPVA	165	5.3	0.150	yes

**Table 3:** Processed materials and their experimentally determined glass transition temperatures, melting temperatures and minimum extrusion temperatures.

Material	Suitable as Filament?	Processable in Hot-End?	T_g_ [°C]	T_m_ [°C]	Min. Extrusion Temperature [°C]	Cuboid Printed?
EPO	no	yes	56.6	-	130	yes
EVA	no	yes	−29.9	85.0 ^a^	85	yes
PEG	no	yes	-	60.4 ^b^	58	no
PLGA	yes	yes	53.7	-	145	yes
PVPVA	no	yes	111.7	-	165	yes

a: End temperature of the characteristic broad melting range of EVA; b: melting peak temperature.

**Table 4:** Summary of the determined residence time distributions conducted with PVPVA at 165 °C and 15 mg min^−1^ with theophylline as a tracer (av ± sd, *n* = 3).

Hydrodynamic Residence Time [min]	Mean Residence Time [min]	t_10_ Quantile [min]	t_50_ Quantile [min]	t_90_ Quantile [min]	Bodensteinnumber [−]
82	93.2 ± 0.9	81.2 ± 1.3	88.9 ± 0.7	99.1 ± 0.6	1.1 ± 0.1

## Data Availability

Data is contained within the article.

## Sample Availability

Samples of the compounds are not available from the authors.
